# QuickStats

**Published:** 2014-04-11

**Authors:** FROM THE NATIONAL CENTER FOR HEALTH STATISTICS

**Figure:**
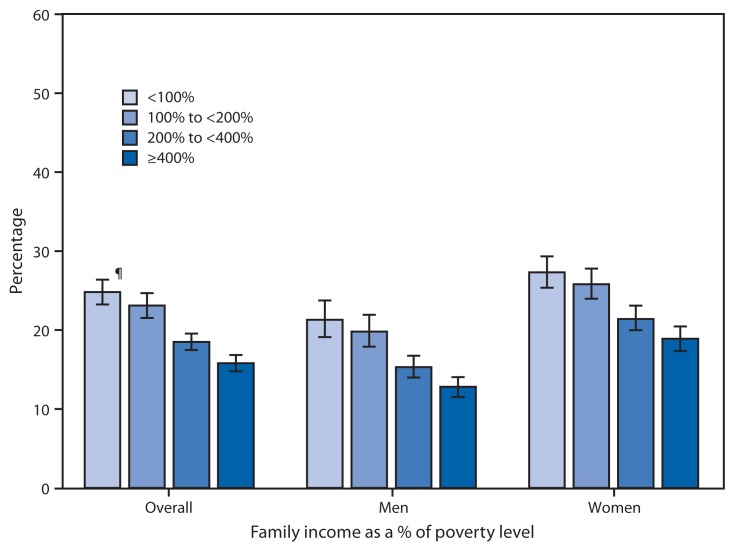
Percentage of Men and Women Who Regularly Had Insomnia or Trouble Sleeping,^*^ by Family Income as a Percentage of Poverty Level^†^ — National Health Interview Survey,^§^ United States, 2012 ^*^Respondents were asked, “During the past 12 months, have you regularly had insomnia or trouble sleeping?” ^†^ Poverty level was based on family income and family size, using U.S. Census Bureau poverty thresholds for 2011. Family income was imputed when information was missing, using multiple imputation methodology. ^§^ Estimates are based on household interviews of a sample of the civilian, noninstitutionalized U.S. adult population. ^¶^95% confidence interval.

During 2012, the percentage of adults aged ≥18 years who reported that they regularly had insomnia or trouble sleeping during the past 12 months ranged from 15.8% for those with family incomes ≥400% of the poverty level to 24.8% for those with family incomes <100% of the poverty level. For both men and women, the percentage who regularly had insomnia or trouble sleeping decreased as family income increased. At every family income level, women were more likely than men to have had insomnia or trouble sleeping.

**Source:** National Health Interview Survey, 2012. Available at http://www.cdc.gov/nchs/nhis.htm.

**Reported by:** Mary Ann Bush, MS, mbush@cdc.gov, 301-458-4130.

